# G-CSF Protects Human Brain Vascular Endothelial Cells Injury Induced by High Glucose, Free Fatty Acids and Hypoxia through MAPK and Akt Signaling

**DOI:** 10.1371/journal.pone.0120707

**Published:** 2015-04-07

**Authors:** Jingjing Su, Houguang Zhou, Yinghong Tao, Jingchun Guo, Zhuangli Guo, Shuo Zhang, Yu Zhang, Yanyan Huang, Yuping Tang, Qiang Dong, Renming Hu

**Affiliations:** 1 Department of Neurology, Shanghai Ninth People's Hospital, Shanghai Jiao Tong University, Shanghai, 200011, China; 2 Department of Geriatric Neurology, Huashan Hospital, Fudan University, Shanghai, 200040, China; 3 Department of General Medicine, Ouyang Hospital, Hongkou District, Shanghai, China; 4 State Key Laboratory of Medical Neurobiology, Department of Neurobiology, School of Basic Medical Science, Shanghai Medical College, Fudan University, Shanghai, 200032,China; 5 Department of Emergency Neurology, the Affiliated Hospital of Medical College Qingdao University, Qingdao, 266100, China; 6 Department of Endocrine, Huashan Hospital, Fudan University, Shanghai, 200040, China; 7 Department of Neurology, Huashan Hospital, Fudan University, Shanghai, 200040, China; Fraunhofer Institute for Cell Therapy and Immunology, GERMANY

## Abstract

Granulocyte-colony stimulating factor (G-CSF) has been shown to play a neuroprotective role in ischemic stroke by mobilizing bone marrow (BM)-derived endothelial progenitor cells (EPCs), promoting angiogenesis, and inhibiting apoptosis. Impairments in mobilization and function of the BM-derived EPCs have previously been reported in animal and human studies of diabetes where there is both reduction in the levels of the BM-derived EPCs and its ability to promote angiogenesis. This is hypothesized to account for the pathogenesis of diabetic vascular complications such as stroke. Here, we sought to investigate the effects of G-CSF on diabetes-associated cerebral vascular defect. We observed that pretreatment of the cultured human brain vascular endothelial cells (HBVECs) with G-CSF largely prevented cell death induced by the combination stimulus with high glucose, free fatty acids (FFA) and hypoxia by increasing cell viability, decreasing apoptosis and caspase-3 activity. Cell ultrastructure measured by transmission electron microscope (TEM) revealed that G-CSF treatment nicely reduced combination stimulus-induced cell apoptosis. The results from fluorescent probe Fluo-3/AM showed that G-CSF greatly suppressed the levels of intracellular calcium ions under combination stimulus. We also found that G-CSF enhanced the expression of cell cycle proteins such as human cell division cycle protein 14A (hCdc14A), cyclinB and cyclinE, inhibited p53 activity, and facilitated cell cycle progression following combination stimulus. In addition, activation of extracellular signal-regulated kinase1/2 (ERK1/2) and Akt, and deactivation of c-Jun N terminal kinase (JNK) and p38 were proved to be required for the pro-survival effects of G-CSF on HBVECs exposed to combination stimulus. Overall, G-CSF is capable of alleviating HBVECs injury triggered by the combination administration with high glucose, FFA and hypoxia involving the mitogen-activated protein kinases (MAPK) and Akt signaling cascades. G-CSF may represent a promising therapeutic agent for diabetic stroke.

## Introduction

Endothelial progenitor cells (EPCs) are a subtype of stem cells with high proliferative and differentiating potential, mainly derived from bone marrow (BM) and peripheral blood (PB) [1]. EPCs express several cell surface markers that differ depending on their source, which allows for easy identification. BM-derived EPCs are immature cells that express CD133 (early hematopoietic stem cell marker), CD34 (progenitor cell marker) and VEGFR-2 (endothelial marker). In contrast, PB-derived EPCs are more mature cells and thus express a variety of endothelial lineage markers such as CD31 (platelet endothelial cell adhesion molecule-1) and CD146 (melanoma cell adhesion molecule) in addition to high expression of VEGFR-2, CD34 and low expression of CD133 [1–3]. The general consensus is that EPCs are mobilized from BM and released into PB subsequently homed to the sites of vascular injury where they differentiate into mature endothelial cells, and ultimately participate in angiogenesis and vascular repair [4–6]. Therefore, EPCs are essential for the maintenance of endothelial integrity.

It has been convincingly documented that diabetes, a metabolic disorder characterized by chronic hyperglycemia and hyperlipemia [7], results in impaired mobilization of BM-derived EPCs and there is a significant reduction in the circulating pool of BM-derived EPCs [8]. Barthelmes et al. reported that streptozotocin-induced diabetic mice failed to mobilize the BM-derived Lin^-^/VEGF-R2^+^ EPCs soon after the onset of diabetes [9]. Recent results from animal experiments showed that diabetic BM displays pathological changes including microangiopathy and neuropathy, which may account for impaired mobilization of BM-derived EPCs in diabetes [10]. Intriguingly, it has been clearly demonstrated that diabetic mice have impaired phosphorylation of BM endothelial nitric oxide synthase (eNOS), which results in reduced EPCs mobilization and low levels of circulating EPCs subsequently [11]. Meanwhile, several clinical trials have also reported that diabetic patients have decreased number of BM-derived EPCs by flow cytometric analysis of cell surface markers including CD133, CD34 and VEGFR-2 [12, 13]. However, there have also been conflicting reports of transient early increments in circulating EPCs occurring in response to acute stroke, hypothesized to promote the repair of damaged vessels [14]. Diabetes has been shown to impair mobilization of BM-derived EPCs and contribute towards defective EPC function. Gallagher et al. found that the EPCs from diabetic mice were unable to home to the injured tissue due to diminished expression of stromal cell-derived factor-1α (SDF-1α), a chemokine that mediated BM-derived EPCs recruitment and homing [11]. Other studies have also shown that human EPCs from diabetic patients exhibit impaired ability to proliferate, adhere and revascularize as a result of increased NAD(P)H oxidase-dependent superoxide production and reduced nitric oxide (NO) bioavailability [15]. Collectively, defective mobilization and function of BM-derived EPCs are believed to be predominantly responsible for the development and progression of diabetic vascular complications such as stroke [9, 11, 16]. It has been established that diabetic patients have a higher incidence of stroke than non-diabetic subjects, and stroke becomes a leading cause of fatality in diabetic patients [17]. Disappointingly, the presence of diabetes confers poor clinical prognosis on diabetic stroke patients and greatly enhances therapeutic difficulties for them [18]. In this regard, making endeavors to searching neuroprotective agents for diabetic stroke is considered to be of great value.

Granulocyte-colony stimulating factor (G-CSF) belongs to a member of the hematopoietic growth factor family. It has been widely used to treat neutropenia in clinical practice due to its properties of promoting mobilization, proliferation and differentiation in hematopoietic progenitor cells [19]. However, growing evidence has suggested that G-CSF has some important non-hematopoietic functions in central nervous system. G-CSF receptor (G-CSFR) as a major G-CSF effector has been found to be universally expressed on various brain cells including neurons and glial cells, and can be largely induced in response to external stimuli such as ischemia and trauma [20]. Recent experimental studies displayed the recovery effects of G-CSF on neurodegenerative diseases such as Alzheimer's disease and Parkinson's disease [21, 22]. More importantly, it has also been frequently described that exogenous G-CSF administration can protect against ischemic rats brain through mobilization of BM-derived progenitor cells, promotion of angiogenesis and neuronal differentiation, and inhibition of apoptosis and inflammation [23–26]. On this basis, combined with our mentioned views regarding the impaired BM-derived EPCs mobilization and angiogenesis in diabetic patients including diabetic stroke [9, 11, 16], we hypothesized that G-CSF may have capacity to exert neuroprotective effects on diabetic stroke.

To test this hypothesis, in the present study, we adopted a newly characterized *in vitro* model of cerebrovascular injury using combination stimulus of high glucose, free fatty acids (FFA) and hypoxia on cultured human brain vascular endothelial cells (HBVECs) to mimic the diabetic cerebrovascular endothelial damage, address the role of G-CSF in this model and dissect the molecular mechanism underlying its function. We found that G-CSF attenuated the combination stimulus-mediated vascular endothelial injury through a series of intracellular pathways including up-regulation of cycle proteins, acceleration of cell cycle process, activation of extracellular signal-regulated kinase1/2 (ERK1/2) and Akt, and deactivation of c-Jun N terminal kinase (JNK) and p38.

## Materials and Methods

### Materials

G-CSF was purchased from Chugai Pharmaceutical Co Ltd (Chuo-ku, Tokyo, Japan). HBVECs were obtained from ScienCell Company (Carlsbad, CA, USA). The Cell Counting Kit-8 (CCK-8) was from Dojindo Molecular Technologies (Gaithersburg, MD, Japan). Caspase-3 activity detection kit was purchased from Millipore (Billerica, MA, USA). Calcium ions fluorescent probe Fluo-3/AM, anti-hCdc14A (human cell division cycle protein 14A) antibody and cell fluorescence dyes (DAPI and rhodamine 6G) were all from Sigma (St. Louis, MO, USA). Annexin V/propidium iodide double-staining assay kit was from BD Company (Franklin L., NJ, USA). Anti-cyclinB, anti-cyclinD, anti-cyclinE and anti-p53 antibodies, horseradish peroxidase (HRP)-conjugated secondary antibody, PD98059 for ERK1/2 inhibitor, SP600125 for JNK inhibitor and SB203580 for p38 inhibitor were all from Cell Signaling Technology (Beverly, MA, USA). Dual Detect ELISA (Enzyme linked immunosorbent assay) Kit for ERK1/2, Akt and p38 were from Millipore (Billerica, MA, USA) and JNK ELISA kit from RayBiotech (Norcross, GA, USA). LY294002 for Akt inhibitor from Calbiochem (La Jolla, CA, USA).

### Cell Cultures and Treatments

HBVECs were cultured in fibronectin-coated dishes in endothelial cell medium at 37°C under 5% CO_2_ and 95% air [27]. The *in vitro* model of HBVECs injury with combination stimulus of high glucose, FFA and hypoxia was carried out as previously described [28]. Our prior dose- (10, 15, 25 mM for high glucose; 50, 100, 200 μM for FFA) and time-dependent (24, 48, 72 h for high glucose and FFA) experiments have demonstrated that the combinatorial exposure of high glucose (25 mM) and FFA (200 μM) to HBVECs for 72 h resulted in maximal inhibitory effects on cellular function [28]. In our present study, baseline oxygen and glucose-containing media were removed from the dishes and kept for future use. Cells were washed three times with PBS, incubated with glucose (25 mM) and FFA (200 μM) for 48 h prior to transfer into a hypoxic chamber containing 85% N_2_, 10% H_2_, and 5% CO_2_ for an additional 24 h (<0.1% O_2_). The combination stimulus was terminated at 72 h by removing the cell cultures from the hypoxic chamber, washing the cultures with PBS, adding the original media and re-incubating the cell cultures in a normoxic incubator [28].

For drug treatments, various concentrations of G-CSF (1, 10, 100, 1000 nM) were added into dishes for the different time. For the pretreatment with G-CSF, G-CSF (100 nM) was applied 12 h before and the duration of combination stimulus. To investigate the effects of signaling pathway inhibitors, PD98059 (30 nM), LY294002 (20 μM), SP600125 (30 nM) and SB203580 (30 nM) were added into cells 30 min before and subsequent exposure to combination stimulus.

### Cell Viability Assay

Cell viability was analyzed by WST-8 assay with Cell Counting Kit-8 (CCK-8) based on the amount of colored formazan produced by dehydrogenase reduction in viable cells. The cells were grown on 96-well plates. CCK-8 (10 μl) solution was added to each well at 37°C for 1 h to form water dissoluble formazan. The absorbance at 450 nm was determined using a microplate reader (Synergy H1, BIO-TEK Instruments, Minneapolis, MN). Results were averaged from three independently repeated experiments.

### Caspase-3 Activity

Caspase-3 activity was measured with the caspase-3 colorimetric activity assay kit. Briefly, the cell lysates were clarified by centrifugation and protein levels in the supernatant were quantitated with a BCA Protein Assay Kit (Thermo Scientific, Rockford, IL). The protein (100 μg) from each sample was loaded into 96-well plates and the culture was incubated with the caspase-3 substrate (10 μl) provided by the kit at 37°C in the dark for 2 h. The absorbance was measured using a fluorescence microplate reader (Synergy H1, BIO-TEK Instruments, Minneapolis, MN) with excitation at 405 nm and emission at 505 nm. Results were averaged from three independently repeated experiments.

### Annexin V/propidium Iodide Double-Staining Assay for Cell Apoptosis

Cell apoptosis was analyzed by Annexin V/propidium iodide (PI) double-staining assay using flow cytometry (EPICS ALTRA, Beckman Coulter Inc., Miami, FL) according to the manufacturer’s instruction. Briefly, cells were trypsinized and washed with serum-containing media. Cells were collected by centrifugation, resuspended in binding buffer (500 μl) with Annexin V-FITC and PI (50 μg/ml), and incubated at room temperature in the dark for 5 min. Annexin V-FITC binding was analyzed by flow cytometry with an FITC signal detector at 530 nm emission, and PI staining was detected with the phycoerythrin emission signal detector. The following controls were used to set up compensation and quadrants: unstained cells, cells stained with Annexin V-FITC (no PI), and cells stained with PI (no Annexin V-FITC). The percentage of Annexin V-FITC/PI double positive cells was calculated. Three independently repeated experiments were conducted.

### Reverse Transcriptase-polymerase Chain Reaction

VEGFR-2 mRNA expression was determined by quantitative reverse transcriptase-polymerase chain reaction (RT-PCR). Total RNA was prepared from cultured cells using TRIzol reagent (Invitrogen, Grand Island, NY, USA). cDNA was generated using RT-PCR kit (TaKaRa, Otsu, Shiga, Japan). The following primers were used: VEGFR-2 forward 5ˊ-GGTCGCATGAACATGA AGAA-3ˊ and reverse 5ˊ-TTGGTAGGGTTTGTAAGGAC-3ˊ, and β-actin forward 5ˊ-CCTCTATG CCAACACAGTGC-3ˊ and reverse 5ˊ-GTACTCCTGCTTGCTGATCC-3ˊ. The reaction was performed using the ABI 9700 PCR system (Applied Biosystems, Foster City, CA) in 20 μl of reaction mixture. Cycling conditions included 30 cycles with denaturation at 94°C for 30 s, annealing at 55°C for 30 s and PCR product extension at 72°C for 1 min. PCR products were detected by 1.3% agarose gel electrophoresis and visualized using Ethidium Bromide staining. These experiments were independently performed three times. Statistical analysis of cell densitometry was performed using TotalLab v2.01 image analysis software (GE Healthcare, Waukesha, WI).

### Cell Ultrastructure Measurement

The cell ultrastructure was measured by transmission electron microscope (TEM). Briefly, the cells were fixed with 2.5% glutaraldehyde and immersed in 1% osmium tetroxide for 2 h, followed by washing in PBS for 15 min. Subsequently, they were dehydrated with acetone, embedded for 2 h, heated at 60°C for 48 h and cut into 50 nm pieces. The sections were dyed with acetic acid uranium and lead, and observed under TEM (Tecnai G2 Spirit, FEI Corp., Hillsboro, OR). The cell ultrastructure was determined from 25 randomly-chosen fields in each sample and results were averaged from three independent experiments.

### Detection of Intracellular Calcium Ions by Fluo-3/AM

Intracellular calcium ions were detected using the fluorescent probe Fluo-3/AM. Following various treatments, cells were incubated with Fluo-3/AM stock solution (final concentration in the mixture 5 μM) at 37°C in the dark for 10 min following three washes with PBS. Fluorescence photomicrographs of cells on glass-bottom dishes were taken using a Nikon Diaphot inverted microscope equipped with a 100W Xenon lamp (Nikon, Tokyo, Japan). The fluorescence intensity was quantified using the fluorescence microplate reader (Synergy H1, BIO-TEK Instruments, Minneapolis, MN) at an excitation of 485 nm and an emission of 535 nm. Results were averaged from three independent experiments.

### Western Blotting

Protein expression was detected by western blotting analysis. Following various treatments, cells were scraped in ice-cold lysis buffer and then clarified by centrifugation at 13,000 g for 25 min. The protein concentration was determined using BCA kit (Thermo Fisher Scientific, Waltham, MA). A total of 30 μg protein was separated by 8% SDS-PAGE and thereafter transferred onto a polyvinylidene difluoride membrane (Roche Applied Science, Mannheim, Germany). The membranes were incubated with anti-hCdc14A (1:1000), anti-cyclinB (1:2000), anti-cyclinD (1:1500), anti-cyclinE (1:1000) or anti-p53 (1:2000) antibodies for 2 h and HRP-conjugated secondary antibody (1:1000) for 1 h. Blotted proteins were tested via an enhanced chemiluminescence assay. Quantification of protein bands was determined by densitometry using TotalLab v2.01 image analysis software (GE Healthcare, Waukesha, WI). Results were averaged from three independent experiments.

### Cell Cycle Determination

Cell cycle process was investigated by propidium iodide (PI, BD Biosciences, Rockville, MD) staining and flow cytometry according to the manufacturer’s protocol. Briefly, cells were fixed in ice-cold 70% ethanol for 1 h, washed again with PBS, and incubated with RNase A (1 mg/ml) at 37°C for 30 min. Then the cells were suspended in 0.5 mL of PI/RNase Staining Buffer and incubated for 15 min at room temperature. Finally, stained cells were analyzed by flow cytometry (EPICS ALTRA, Beckman Coulter Inc., Miami, FL). Unstained cells were used as controls. The percentage of cells in each cell cycle phase was analyzed using Multicycle software (Beckman Coulter Inc., Miami, FL). Three independently repeated experiments were conducted.

### Confocal Microscopy

Alterations in phosphorylated signaling proteins and F-actin with G-CSF treatment following combination stimulus were detected by confocal microscopy. The cells were seeded on a 6-well plate containing an aseptic cover glass treated with polylysine (Sinopharm Chemical Reagent Co., Ltd, Shanghai, China) and washed three times in PBS. They were fixed with 4% paraformaldehyde (Sinopharm Chemical Reagent Co., Ltd, Shanghai, China) for 30 min and then permeabilized with 0.5% Triton-X in PBS for 30 min. Followed by three washes, cells were incubated with the anti-phospho-ERK1/2, anti-phospho-Akt, anti-phospho-JNK and anti-phospho-p38 antibodies (diluted 1:200) (Cell Signaling Technology, Beverly, MA, USA). After washing three times, they were treated with the CY3-labled secondary antibody (Jackson ImmunoResearch, West Grove, PA). F-actin was stained with the cytoskeletal staining agent rhodamine 6G at room temperature in the dark for 40 min. Cell nucleus was stained with the DAPI staining agent at 4°C for 1 min. Fluorescent labeling was visualized under a Leica TCS SP5 confocal microscope (Leica Microsystems Heidelberg GmbH, Germany). The phosphorylated proteins were detected in 3 randomly-chosen fields and F-actin was detected in 100 randomly-chosen cells in each sample. Three independently repeated experiments were conducted.

### ELISA

The effect of combination stimulus at different time points on phosphorylated signaling proteins was evaluated using ELISA. Cells were cultured in 96-well plate and fixed with 4% paraformaldehyde (Sinopharm Chemical Reagent Co., Ltd, Shanghai, China) for 20 min followed by washing three times in TBST. Then they were blocked in 10% BSA at room temperature for 1 h. Cells were simultaneously incubated with anti-ERK1/2 (total) and anti-phospho-ERK1/2 antibodies, or anti-Akt (total) and anti-phospho-Akt antibodies, or anti-JNK (total) and anti-phospho-JNK antibodies, or anti-p38 (total) and anti-phospho-p38 antibodies. The levels of both the total target proteins and the phosphorylated proteins were measured simultaneously in a single well using a double immunoenzymatic labeling procedure, either HRP or alkaline phosphatase (AP), and two spectrally distinct fluorogenic substrates for HRP or AP. The fluorescence of the phosphorylated proteins was normalized to the total protein in each well thus reducing well-to-well variations. Three independently repeated experiments were conducted.

### Data Analysis

Results were expressed as mean±SD. Statistical analyses were conducted using one-way ANOVA following post-hoc tests for multiple comparisons. For comparisons of two groups alone, Unpaired Student’s t-tests were applied. P<0.05 was considered to be statistically significant.

## Results

### G-CSF Enhances Cell Viability and Prevents Apoptosis Induced by Combination with High Glucose, FFA and Hypoxia on HBVECs

**Fig 1:**
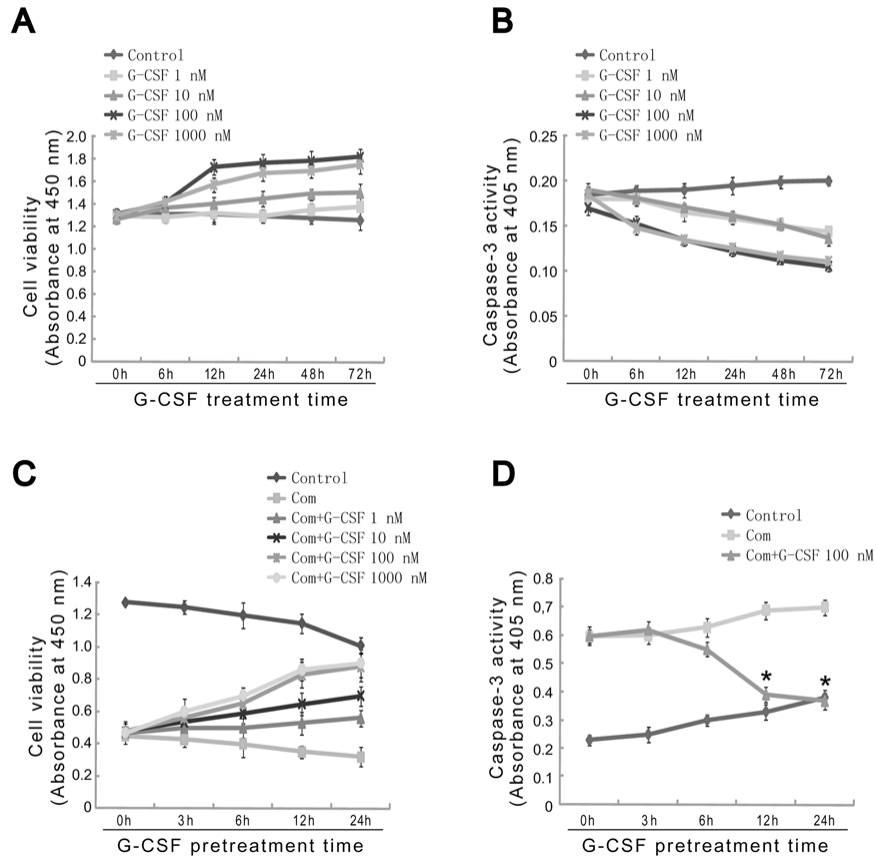
Concentration- and time-dependent effects of G-CSF on cell viability and caspase-3 activity in cultured HBVECs. (A, B) Cells were treated with various concentrations of G-CSF (1, 10, 100, 1000 nM) for different time (6, 12, 24, 48, 72 h). (C) Pretreatment with G-CSF (1, 10, 100, 1000 nM) for different time (3, 6, 12, 24 h) followed by combination stimulus and cell viability was observed. G-CSF promoted cell viability in a dose- and time-dependent manner in cultured HBVECs after combination stimulus. (D) Cells were pretreated with G-CSF (100 nM) for different time (3, 6, 12, 24 h) followed by combination stimulus. The results showed that G-CSF preincubation followed by combination stimulus reduced caspase-3 activity in a time-dependent manner compared with combination stimulus alone. With the combination stimulus, cells were exposed to glucose (25 mM) and FFA (200 μM) for 48 h, followed by an additional 24 h in the presence of hypoxia. Results were obtained from three independently repeated experiments. *p<0.05 vs. Com at the corresponding time points. Com: combination stimulus.

**Fig 2:**
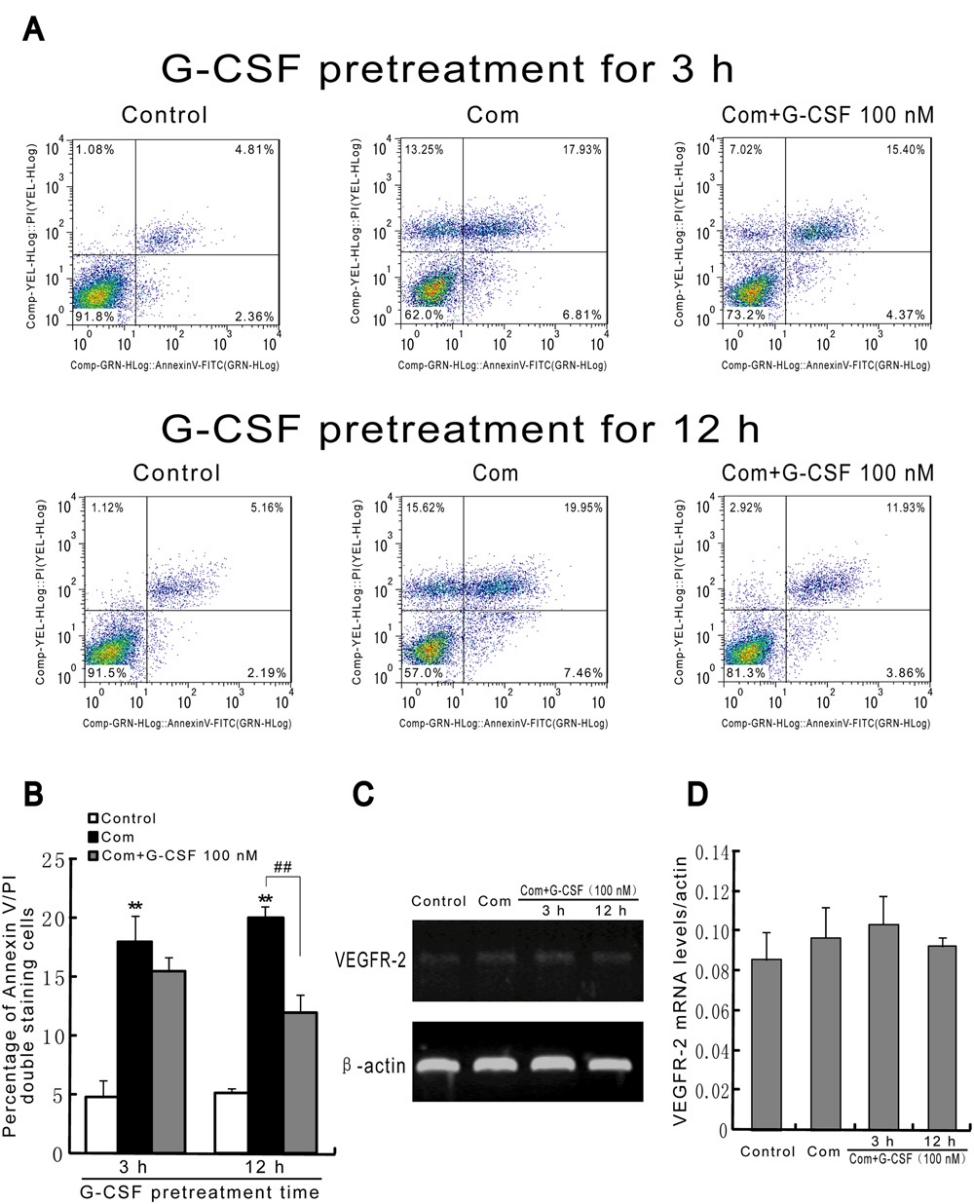
G-CSF preincubation followed by combination stimulus reduces cell apoptosis analyzed by flow cytometry in HBVECs, but has no significant effect on the mRNA expression of VEGFR-2. G-CSF (100 nM) was applied 3 h and 12 h before and during combination stimulus. (A) Flow cytometry graphical data for cell apoptosis. (B) Quantitative analysis of cell apoptosis by flow cytometry. (C) VEGFR-2 mRNA expression was analyzed. (D) The relative density of VEGFR-2 mRNA was calculated to that of β-actin. Results were obtained from three independently repeated experiments. **p<0.01 vs. control. ## p<0.01 for Com + G-CSF vs. Com. Com: combination stimulus.

To investigate the exact roles of G-CSF in HBVECs injury induced by high glucose, FFA and hypoxia, in the present study, we first intended to determine the effects of G-CSF on cell viability and caspase-3 activity in the cultured HBVECs. Cells were treated with various concentrations of G-CSF (1, 10, 100, 1000 nM) for different time points (6, 12, 24, 48, 72 h). As shown in Fig 1A and 1B, G-CSF induced a dose- and time-dependent increase in cell viability and decrease in caspase-3 activity. We next addressed the alterations in cell viability and apoptosis induced by combination of high glucose, FFA and hypoxia on HBVECs after G-CSF pretreatment. The results showed that preincubation of G-CSF for the different periods markedly increased cell viability in a concentration- and time-dependent manner following the combination stimulus, with nearly similar G-CSF effects at concentrations of 100 nM and 1000 nM (Fig 1C). Subsequently, we found that G-CSF (100 nM) pretreatment significantly reduced caspase-3 activity and cell apoptosis following combination stimulus in cultured HBVECs compared with combination stimulus alone at the corresponding time points (Fig 1D, Fig 2A and 2B). Indeed, the pretreatments with G-CSF at 12 h and 24 h appeared to have nearly identical protective effects (Fig 1C and 1D). However, G-CSF (100 nM) administration at 3 h and 12 h following combination stimulus showed no significant effect on mRNA expression of VEGFR-2, a cell surface marker of HBVECs, compared with combination stimulus alone, which suggests that G-CSF is unable to induce HBVECs differentiation under our experimental conditions (Fig 2C and 2D). These results indicated that G-CSF can promote cell viability and inhibit apoptosis triggered by combination addition with high glucose, FFA and hypoxia to HBVECs in a dose- and time-dependent manner. Based on the above results, in the following experiments, we focused on examining the effects of G-CSF pretreatment on cell injury at the concentration of 100 nM for 12 h.

### Effects of G-CSF on Cell Ultrastructure by TEM after Combination Stimulus

**Fig 3:**
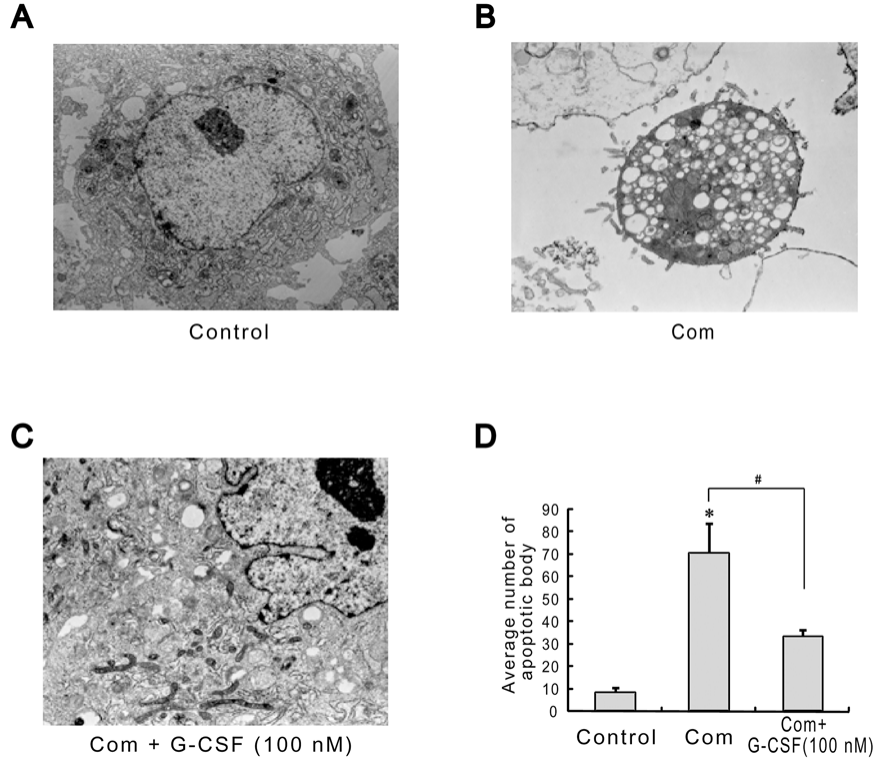
Alterations in cell ultrastructure examined by TEM during the addition of G-CSF (100 nM) to HBVECs for 12 h before and throughout the combination stimulus. (A) The intact cell ultrastructure under normal condition. (B) Combination stimulus led to organelles loss and apoptotic body formation. (C) G-CSF remarkablely ameliorated these injured changes, such as the significant decrease in apoptotic bodies. (D) Quantitative assay of apoptotic bodies. Twenty-five randomly-chosen fields were observed in each sample. Three independently repeated experiments were conducted. *p<0.05 vs. control. #*p*<0.05 for Com + G-CSF vs. Com. Com: combination stimulus. Original magnification: 90,000×.

To further determine the anti-apoptotic effects of G-CSF on the combination stimulus-induced injury, we used TEM to monitor the changes in cell ultrastructure in cultured HBVECs. Our data indicated that cell ultrastructure remained intact under normal condition (Fig 3A). But combination treatment enabled cells to manifest the obvious ultrastructural changes, such as organelles loss and apoptotic body formation with a great deal of autophagic vacuole (Fig 3B). Importantly, pretreatment with 100 nM G-CSF for 12 h prior to and during the combination stimulus exquisitely rescued these changes, characterized by the significant reduction in apoptotic body (Fig 3C). Quantitative assay of apoptosis showed that combination stimulus resulted in high levels of apoptotic bodies (69.7 ± 13.5) relative to control (7.7 ± 2.1). G-CSF administration following combination stimulus led to a marked reduction in the number of apoptotic bodies to 32.7 ± 3.1 (*p*<0.05; Fig 3D). These results further presented evidence about the protective effects of G-CSF against combination stimulus insults.

### G-CSF Reduces Combination Stimulus-induced Calcium Ion Levels Measured by Fluorescent Probe Fluo-3/AM

**Fig 4:**
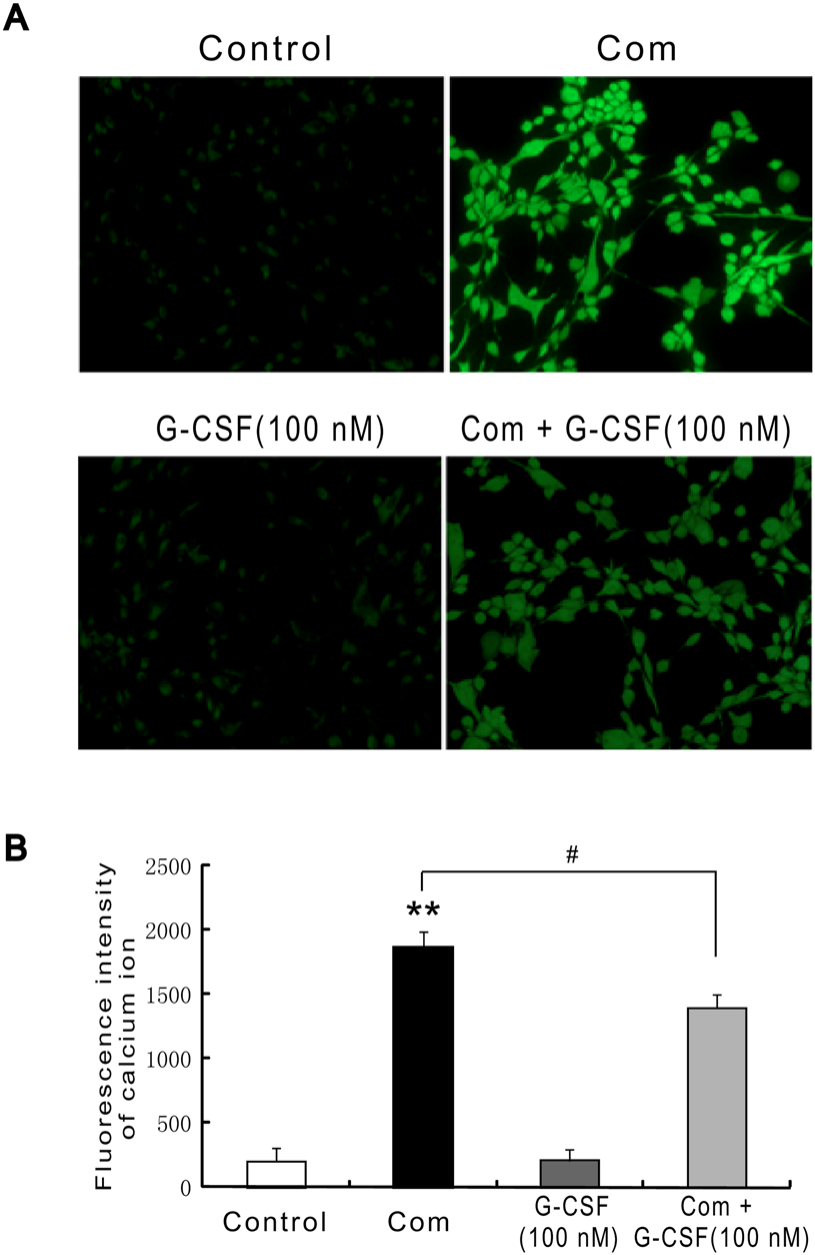
G-CSF pretreatment reduces combination stimulus-induced calcium ion levels measured by fluorescent probe Fluo-3/AM. G-CSF (100 nM) was added to the medium 12 h before and subsequent exposure to combination stimulus. (A) Combination stimulus induced high fluorescence intensity relative to the normal condition or G-CSF alone. Importantly, G-CSF pretreatment greatly reversed these changes. (B) Quantitative analysis about fluorescence intensity. Three independently repeated experiments were conducted. **p<0.01 vs. normal control. #p<0.05 for Com + G-CSF vs. Com. Com: combination stimulus.

It is well known that the concentration of intracellular calcium ions is closely related to the severity of cell injury [29]. To investigate the effects of G-CSF on the levels of intracellular calcium ions, in this study, fluorescent probe Fluo-3/AM was used to label intracellular calcium ions. The results showed that normal condition or the addition of G-CSF alone to HBVECs showed the similar low fluorescence intensity. However, combination stimulus of high glucose, FFA and hypoxia largely induced increase in Fluo-3/AM fluorescence intensity, suggesting this stimulus can result in cell injury. Notably, the addition of 100 nM G-CSF for 12 h before and throughout the combination stimulus greatly suppressed this fluorescence intensity compared with combination stimulus alone (Fig 4A and 4B). These results also showed the potential ability of G-CSF to alleviate combination stimulus-induced cell damage.

### G-CSF Affects the Expression of Cycle Proteins under Combination Stimulus

**Fig 5:**
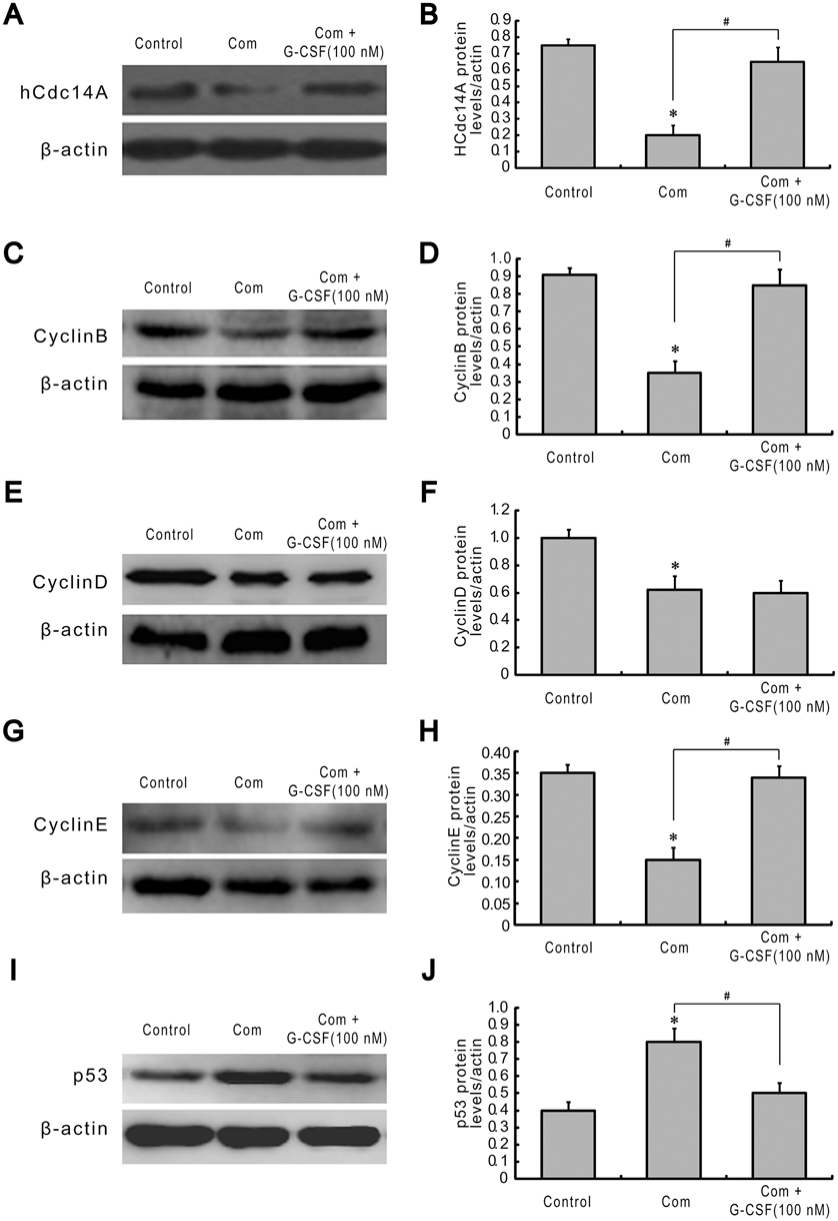
G-CSF pretreatment affects the expression of cycle proteins in HBVECs following combination stimulus. Cells were incubated with G-CSF (100 nM) for 12 h and thereafter exposed to combination stimulus together with G-CSF (100 nM). The protein expression of hCdc14A (A), cyclinB (C), cyclinD (E), cyclinE (G) and p53 (I) was analyzed. The relative density of hCdc14A (B), cyclinB (D), cyclinD (F), cyclinE (H) and p53 (J) was calculated to that of β-actin. The results showed that G-CSF treatment enhanced the expression of hCdc14A, cyclinB and cyclinE, and reduced p53 level, compared to combination stimulus alone. Results were obtained from three independently repeated experiments. *p<0.05 vs. control. #p<0.05 for Com + G-CSF vs. Com. Com: combination stimulus.

It has been shown that cell cycle can closely regulate the processes of cell proliferation and apoptosis [30]. Additionally, many cycle proteins such as hCdc14A, cyclinB, cyclinD, cyclinE and p53 are considered to be the central participants in the regulation of cell cycle events [31, 32]. Herein, we first tested whether G-CSF can effect the changes in these cycle proteins expression under combination stimulus. As shown in Fig 5, compared with normal controls, combination stimulus significantly inhibited the expression of hCdc14A, cyclinB, cyclinD and cyclinE by 0.27-, 0.38-, 0.62-, and 0.43-fold respectively, and increased p53 level 2-fold in HBVECs. However, G-CSF pretreatment for 12 h following combination stimulus markedly reversed these changes, characterized by an increase in hCdc14A, cyclinB and cyclinE expression levels by 3.25-, 2.43-, and 2.27-fold and a 0.63-fold decrease in p53 levels, compared with combination stimulus alone without significant change in cyclinD levels. These findings indicated that G-CSF can up-regulate hCdc14A, cyclinB and cyclinE expression, and depress p53 expression in cultured HBVECs subjected to combination stimulus.

### G-CSF Facilitates Cell Cycle Progression under Combination Stimulus

**Fig 6:**
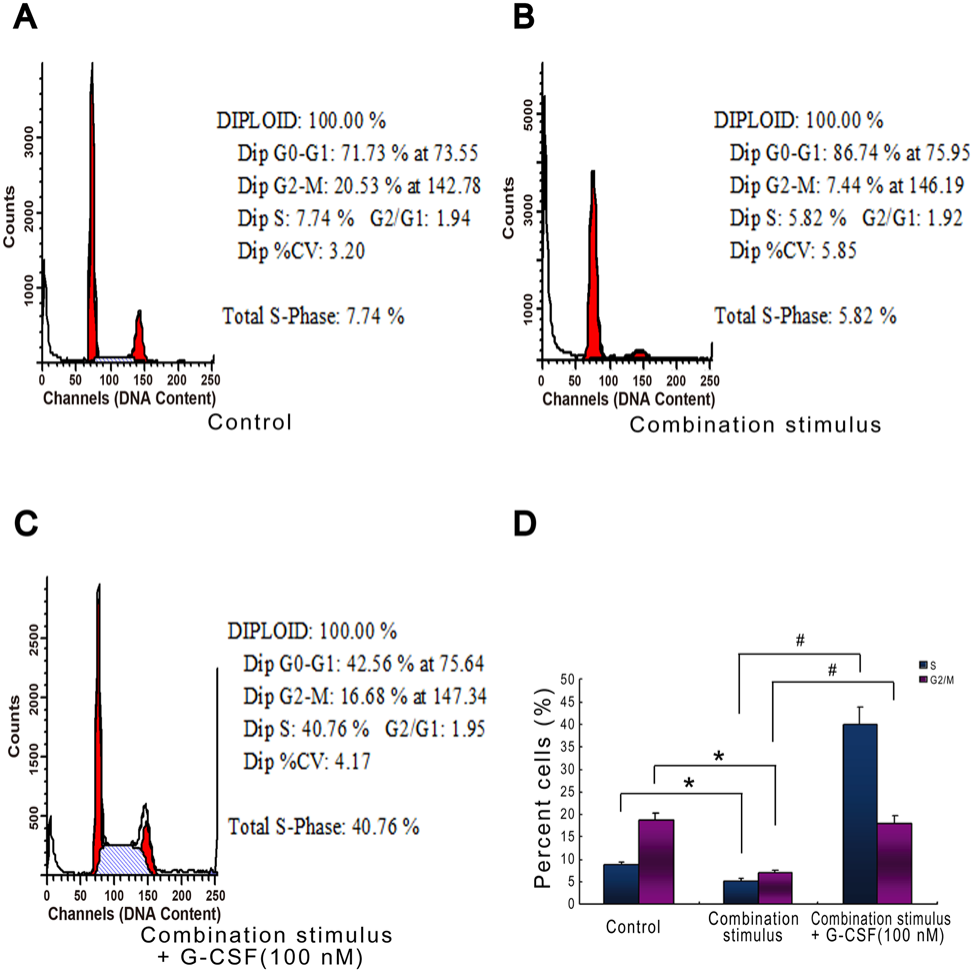
G-CSF facilitates cell cycle progression under combination stimulus in HBVECs. (A-C) Flow cytometry graphical data for cell cycle. (D) Quantitative assay of cell cycle indicated that G-CSF (100 nM) pretreatment largely enhanced the combination stimulus-induced decreases in cell proportion in S and G2/M phases. Three independently repeated experiments were conducted. *p<0.05 for combination stimulus vs. control. #p<0.05 for combination stimulus + G-CSF vs. combination stimulus alone.

**Fig 7:**
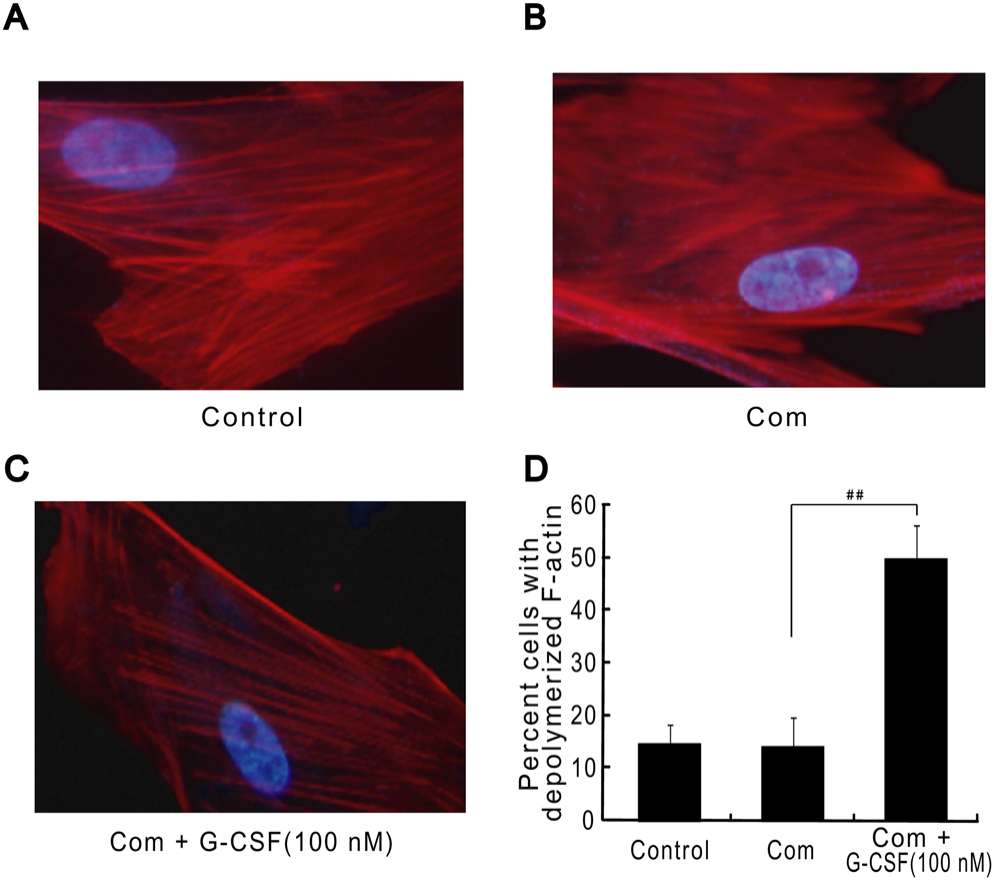
G-CSF pretreatment leads to the formation of depolymerized skeleton structure in HBVECs after combination stimulus by immunofluorescence analysis. (A) The clear filamentary structure in normal cells. (B) The fibrous fracture after combination stimulus. (C) The depolymerized skeleton structure after G-CSF (100 nM) exposure following combination stimulus. Cells were stained for F-actin in red and nuclei with DAPI in blue. (D) Quantitative assay of depolymerized F-actin. One-hundred randomly-chosen cells were observed in each sample and three independently repeated experiments were conducted. ##p<0.01 for Com + G-CSF vs. Com alone. Com: combination stimulus.

Subsequently, we observed the effects of G-CSF on cell cycle process under combination stimulus by flow cytometry assay. Our data revealed that combination stimulus obviously reduced the cell proportion in S and G2/M phases relative to the basal levels. However, G-CSF treatment dramatically improved this cell proportion compared with combination stimulus-treated cells, indicating that G-CSF appeared to facilitate cell cycle process under this damage condition (Fig 6). The results from cytoskeletal observation by immunofluorescence analysis showed that in normal condition, the skeleton protein F-actin displayed clear filamentary structure (Fig 7A). Combination stimulus led to the disruption of normal structure, such as fibrous fracture and disorder (Fig 7B). Importantly, the exposure of G-CSF to cells exhibited depolymerized changes in skeleton protein structure, such as the loosened and clear filamentary structure, which was considered to prepare for mitotic process (Fig 7C). Quantitatively, depolymerized F-actin was detected in 14.7 ± 3.5% and 14 ± 5.6% of normal cells and combination stimulus-treated cells, respectively. G-CSF treatment for 12 h before and throughout the combination stimulus significantly increased the proportion of cells with depolymerized F-actin at 49.7 ± 6.5% compared with combination stimulus alone (*p*<0.01; Fig 7D). Collectively, we presumed that G-CSF can accelerate cell cycle progression and thereby probably influencing cell proliferation and inhibiting apoptosis.

### Roles of Signaling Pathways in the Anti-apoptotic Effects of G-CSF during Combination Stimulus

**Fig 8:**
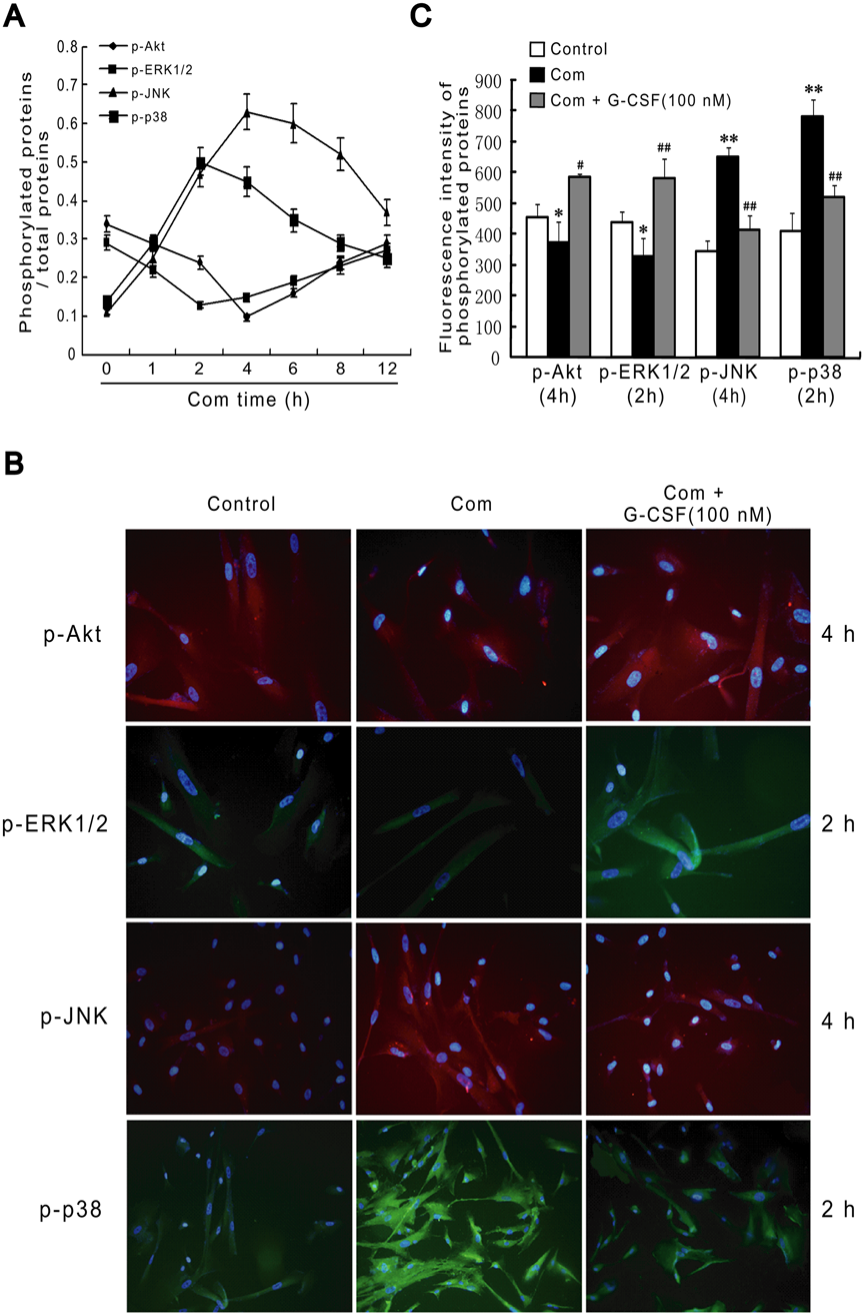
Combination stimulus and combination with G-CSF treatment affect the phosphorylation levels of MAPK and Akt signaling cascades in HBVECs. (A) ELISA showed that continuous combination stimulus up-regulated p-JNK and p-p38 expression whereas down-regulated p-ERK1/2 and p-Akt expression. (B) Immunofluorescence experiments indicated that G-CSF (100 nM) pretreatment for 12 h before and during combination stimulus increased p-ERK1/2 and p-Akt expression at 2 h and 4 h of combination stimulus, respectively, while inhibited p-p38 and p-JNK expression at the same time points. HBVECs were stained for p-Akt and p-JNK in red, p-ERK1/2 and p-p38 in green, and nuclei with DAPI in blue. (C) Quantitative analysis about fluorescence intensity of phosphorylated proteins by immunofluorescence experiments and three randomly-chosen fields were detected. Results were obtained from three independently repeated experiments. *p<0.05 and **p<0.01 vs. control. #*p*<0.05 and ##*p*<0.01 vs. Com. Com: combination stimulus.

Mitogen-activated protein kinases (MAPK) signaling cascades are mainly composed of ERK1/2, JNK and p38. They have been documented to be implicated in cell proliferation and differentiation [33, 34]. Akt pathway has been proved to mediate the pro-survival process following various damages [35]. Therefore, we tested whether these signaling pathways had been involved in the anti-apoptotic activity of G-CSF in cultured HBVECs challenged by the simultaneous addition of high glucose, FFA and hypoxia. We first sought to observe the expression of these signaling pathways under combination stimulus. As described in Fig 8A, cells exposed to the different time points of combination stimulus displayed the induction in phosphorylation of p38 and JNK (p-p38, p-JNK), which peaked at 2 h and 4 h of combination stimulus, respectively, and thereby gradually falling down. In contrast, the continuous stimulus led to the decline in phosphorylation of ERK1/2 and Akt (p-ERK1/2, p-Akt) and these decreases reached their lowest points at 2 h and 4 h of combination stimulus, respectively. Hence, the time-dependent dynamic changes in the expression of these signaling proteins followed by the continuous combination stimulus revealed that the activity of these signaling pathways might participant in the stimulus-triggered insults.

We next examined the effects of G-CSF on phosphorylation levels of MAPK and Akt signaling cascades under combination stimulus. Immunofluorescence experiments showed that when compared with normal controls, the levels of p-ERK1/2 and p-Akt were significantly reduced at 2 h and 4 h of combination stimulus, respectively. The fluorescence intensity of p-ERK1/2 and p-Akt at 2 h and 4 h of combination stimulus decreased to 328.7 ± 61 and 373.3 ± 70.2 from 436 ± 37.6 and 454.3 ± 46.7 in normal controls, respectively. Pretreatment with G-CSF for 12 h before and the duration of combination stimulus dramatically enhanced the fluorescence intensity of p-ERK1/2 and p-Akt (579 ± 66.2 and 583.3 ± 15.3) (*p*<0.05 and *p*<0.01; Fig 8B and 8C). Reciprocally, compared with controls, combination stimulus at 2 h and 4 h increased the fluorescence intensity of p-p38 (783.3 ± 56.9 vs. 409.7 ± 62.1) and p-JNK (649.3 ± 35.2 vs. 342 ± 38.6), respectively. Crucially, G-CSF treatment greatly suppressed these increases (518 ± 42.9 and 414.3 ± 47.2) (*p*<0.01; Fig 8B and 8C). These results suggested that G-CSF treatment activated ERK1/2 and Akt signaling pathways while inhibited JNK and p38 signaling cascades following combination stimulus.

**Fig 9:**
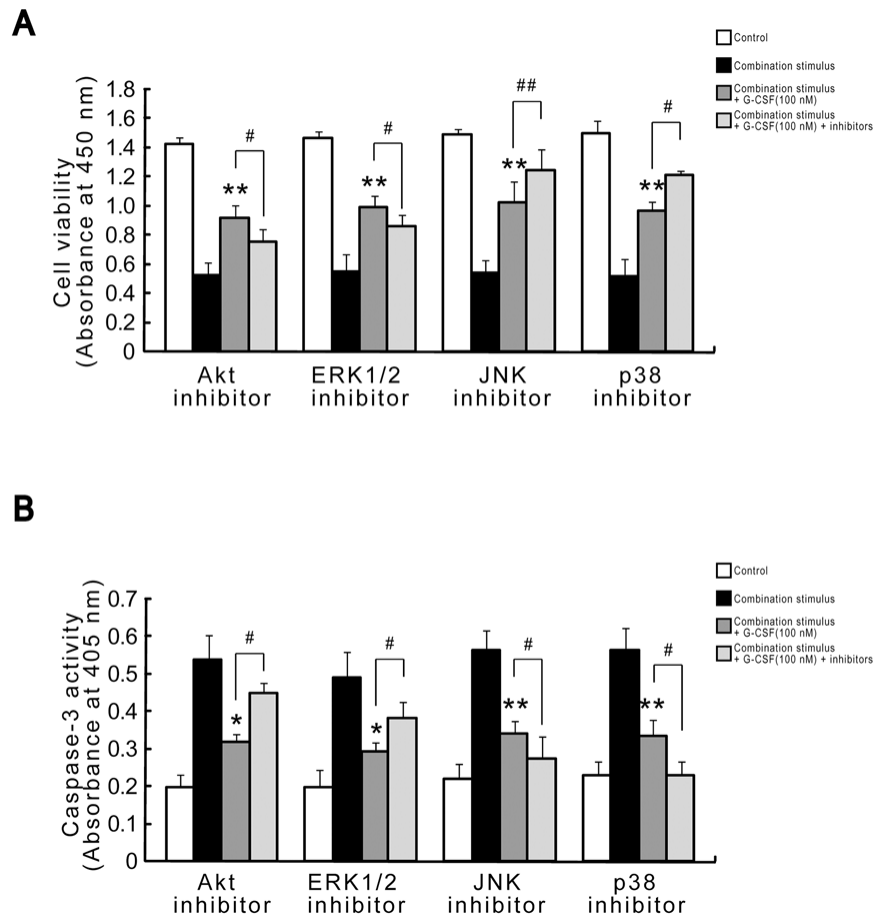
Protective functions of G-CSF in combination stimulus depends on activity of MAPK and Akt signaling cascades. The addition of ERK1/2 or Akt inhibitors (30 nM PD98059, 20 μM LY294002) 30 min before combination stimulus almost completely abrogated the protective functions of G-CSF (100 nM) following combination stimulus by decreasing cell viability (A) and increasing caspase-3 activity (B). However, the administration of JNK or p38 inhibitors (30 nM SP600125, 30 nM SB203580) produced the opposite effects (A, B). Three independently repeated experiments were conducted. *p<0.05 and **p<0.01 vs. combination stimulus. #p<0.05 and ##p<0.01 for combination stimulus + G-CSF + inhibitors vs. combination stimulus + G-CSF.

Lastly, we investigated the involvements of MAPK and Akt signaling cascades in the protection of G-CSF in combination stimulus. We conducted CCK-8 assay and caspase-3 activity measurement. The present data indicated that G-CSF exposure for 12 h prior to and during the combination stimulus effectively prevented the declines in cell viability and increases in caspase-3 activity triggered by the combination stimulus. Surprisingly, the application of ERK1/2 or Akt inhibitors (PD98059, LY294002) 30 min before combination stimulus almost completely abolished the survival-promoting ability of G-CSF following combination stimulus by decreasing cell viability and increasing caspase-3 activity. However, the treatments of JNK or p38 inhibitors (SP600125, SB203580) produced the opposite effects, such as increases in cell viability and decreases in caspase-3 activity (Fig 9). Taken together, our findings demonstrated that the protective functions of G-CSF might at least partially depend on the activation of ERK1/2 and Akt, and the deactivation of JNK and p38 in HBVECs subjected to combination stimulus.

## Discussion

It has been demonstrated that hyperglycemia and hyperlipemia are the predominantly metabolic characteristics of diabetes and hypoxia represents the well-established feature of cerebral ischemia [7, 36]. Hence, we developed an *in vitro* cerebrovascular injury model using combination stimulus of high glucose, FFA and hypoxia on cultured HBVECs to mimic diabetic cerebrovascular damage and test the effects of G-CSF on this type of injury. Previous studies indicated that various types of cells subjected to high glucose and FFA suffered from extensive cellular damage [27, 37]. In this study, we found that the cultured HBVECs showed detrimental responses to the combination stimulus of high glucose, FFA and hypoxia, as evidenced by dramatic losses of cell viability and sharp increases in apoptosis and intracellular calcium ions, suggesting the combination stimulus can lead to cerebrovascular endothelial damage.

G-CSF functions as a potent growth factor for promoting the mobilization, proliferation, homing ability and differentiation of BM-derived EPCs. Findings from cancer animal models suggest that G-CSF promotes tumor growth by increasing mobilization and differentiation of BM-derived EPCs [38]. Both experimental and clinical studies demonstrated that G-CSF administration enhanced mobilization of BM-derived EPCs and improved their ability to home to ischemic myocardium, resulting in improved heart function [8, 39]. Qiu et al. showed that G-CSF was able to promote proliferation of BM-derived EPCs [40]. Collectively, these studies support the essential roles of G-CSF in BM-derived EPC mobilization, proliferation, homing to injury sites and differentiation. G-CSF has also been reported to protect against ischemic brain injury. Both animals and clinical trials displayed the advantageous effects of G-CSF on ischemic stroke [23–26, 41, 42]. Recent studies also showed the strong efficacy of G-CSF in protecting acute and chronic ischemic stroke [41, 42]. Furthermore, the preliminary results from a rat model of diabetic cerebral ischemia have revealed the anti-apoptotic effects of G-CSF on brain tissue [43]. In the present study, we found that treatment of cultured HBVECs with G-CSF before and during the combination stimulus profoundly rescued the damage-induced decrease in cell viability, increase in caspase-3 activity and apoptosis, but had no significant effect on HBVECs differentiation defined by the mRNA expression of cell surface marker VEGFR-2. The latter may be due to HBVECs being terminally differentiated. These results suggest that G-CSF has *in vitro* therapeutic potential for reducing diabetic cerebrovascular injury.

It has been documented that cell cycle acts as the critical regulatory process of cell proliferation and apoptosis. Cell cycle arrest may lead to the occurrence of apoptosis, while the accelerated process accompanies excessive proliferation and tumorigenesis [30]. It has also been proved that the activation of cycle proteins such as cyclinB, cyclinD and cyclinE can accelerate cell cycle process, whereas p53 activity blocks this process [31, 32]. Meanwhile, a recent pilot study from our lab presented evidence of hCdc14A, an important cycle regulatory phosphatase, in speeding up cell cycle progression and enhancing cell proliferation in cultured HBVECs following combination stimulus by overexpression of plasmids and small interfering RNA (siRNA) [28]. Previous studies demonstrated that G-CSF had capability of enhancing cyclinD and cyclinE expression, decreasing p53 activity, and thereby accelerating cell cycle progression in hematopoietic and endothelial cell lineages [40, 44–47]. However, little is known about the potential impacts of G-CSF on cycle proteins expression and cell cycle process in HBVECs exposed to combination stimulus of high glucose, FFA and hypoxia. In the present study, we observed that the exposure of HBVECs to G-CSF treatment following combination stimulus greatly boosted the levels of hCdc14A, cyclinB and cyclinE, lessened p53 and accelerated cell cycle transition, but had no obvious change in cyclinD level. Collectively, from these results we presumed that under our experimental conditions G-CSF achieved a significant promotion in cell proliferation probably through influencing cycle proteins expression and subsequently speeding up cell cycle process.

A body of previous evidence indicated that ERK1/2 and Akt signaling cascades mediated the protection afforded by G-CSF administration in cerebral, myocardial and spinal cord injury *in vivo* and *in vitro* [48–50]. Instead, the inhibition of JNK and p38 activity might be required for the G-CSF efficacy [50]. Here, to clarify whether these signaling pathways were involved in the anti-apoptotic effects of G-CSF on cultured HBVECs, we therefore examined their activity during combination stimulus after G-CSF exposure. The results showed that G-CSF treatment markedly enhanced combination stimulus-induced decreases in p-ERK1/2 and p-Akt levels, and reduced p-p38 and p-JNK activity, indicating that these signaling cascades might be related to the G-CSF neuroprotection. Subsequently, we analyzed the relationship between the activation of these signaling and G-CSF function. We found that pharmacological inhibition of ERK1/2 and Akt pathways significantly attenuated G-CSF-mediated apoptosis delay in HBVECs challenged by the combination stimulus. In contrast, the blockage of JNK and p38 pathways produced opposite effects. These findings reveal that G-CSF plays a beneficial role in combination stimulus-induced injury involving activation of ERK1/2 and Akt, and deactivation of JNK and p38.

ERK3, an atypical MAPK, is suggested to play a role in cell cycle progression and cellular differentiation. Recent study showed that its function is regulated in a cell cycle-dependent manner. Tanguay et al. reported that hCdc14A can bind to the C-terminus of ERK3, reverse its C-terminal phosphorylation and regulate its stability in mitosis [51]. Intriguingly, our current results have verified that G-CSF exposure to HBVECs following combination stimulus largely increased hCdc14A expression and accelerated cell cycle transition. Taken together, we hypothesized that under our experimental conditions G-CSF exhibited its pro-survival effects likely through cell cycle pathway involving hCdc14A and ERK3 activity.

Generally, our present study showed that G-CSF protected HBVECs injury induced by combination addition of high glucose, FFA and hypoxia. In addition, these protective effects at least partially depended on cell cycle regulation, and MAPK, Akt signaling cascades. Here, we highlight the insight that G-CSF might be required for cerebrovascular repair and therapeutic potential in diabetic stroke.
